# Retrieval of a well-established skill is resistant to distraction: Evidence from an implicit probabilistic sequence learning task

**DOI:** 10.1371/journal.pone.0243541

**Published:** 2020-12-10

**Authors:** Teodóra Vékony, Lilla Török, Felipe Pedraza, Kate Schipper, Claire Pleche, László Tóth, Karolina Janacsek, Dezso Nemeth

**Affiliations:** 1 Lyon Neuroscience Research Center (CRNL), INSERM, CNRS, Université Claude Bernard Lyon 1, Lyon, France; 2 Department of Neurology, University of Szeged, Szeged, Hungary; 3 Department of Psychology and Sport Psychology, University of Physical Education, Budapest, Hungary; 4 Institute of Psychology, Université Lumière - Lyon 2, Lyon, France; 5 Centre for Thinking and Learning, Institute for Lifecourse Development, School of Human Sciences, Faculty of Education, Health and Human Sciences, University of Greenwich, London, United Kingdom; 6 Institute of Psychology, ELTE Eötvös Loránd University, Budapest, Hungary; 7 Brain, Memory and Language Research Group, Institute of Cognitive Neuroscience and Psychology, Research Centre for Natural Sciences, Budapest, Hungary; Waseda University, JAPAN

## Abstract

The characteristics of acquiring new sequence information under dual-task situations have been extensively studied. A concurrent task has often been found to affect performance. In real life, however, we mostly perform a secondary task when the primary task is already well acquired. The effect of a secondary task on the ability to retrieve well-established sequence representations remains elusive. The present study investigates whether accessing well-acquired probabilistic sequence knowledge is affected by a concurrent task. Participants acquired non-adjacent regularities in an implicit probabilistic sequence learning task. After a 24-hour offline period, participants were tested on the same probabilistic sequence learning task under dual-task or single-task conditions. Here, we show that although the secondary task significantly prolonged the overall reaction times in the primary (sequence learning) task, access to the previously learned probabilistic representations remained intact. Our results highlight the importance of studying the dual-task effect not only in the learning phase but also during memory access to reveal the robustness of the acquired skill.

## Introduction

Sequence learning is a fundamental function of the brain that underlies the acquisition of motor, cognitive, and social skills [1–4]. These skilled actions, such as driving a car or playing sports, usually become automatic with extensive practice. In everyday life, we generally do not perform these actions in isolation but simultaneously with other actions. The effect of a secondary task on implicit sequence learning has been studied extensively in the last few decades. Evidence for impaired [5–11], intact [4, 7, 9, 12, 13], or even improved performance [6] was found during the acquisition of implicit sequence knowledge. Despite the vast literature on the effect of a secondary task on sequence learning, its effect on the *retrieval* of a well-established skill is rarely studied. In everyday life, we mostly perform a secondary task when the primary task is well-learned. For example, when we learn how to drive, our entire attention is focused on this particular task, and we refrain from other concurrent activities, such as chatting with our co-pilot. However, after mastering this skill, we easily engage in conversations during the primary (driving) task. Therefore, answering whether our performance is affected in such cases is crucial in understanding the effects of a secondary task on real-life performances. Here, we present a study testing the effects of a secondary task on retrieving implicit probabilistic sequence knowledge, which is a crucial aspect of skill-learning.

Using short single-task practice periods and immediate retrieval, early studies found impaired [14] or intact retrieval of sequence knowledge under dual-task conditions [15, 16]. The latter results were often interpreted as evidence for the secondary task affecting only the performance measured at the time of testing (i.e., the expression of knowledge), but not the underlying representations [15]. The fact that the detrimental effects of dual-tasking decrease with practice [17] raises the possibility that sequence knowledge remains intact when experience has already accumulated in the primary task. Results from simple choice-response tasks, without any sequence to learn, also imply that an already automatized behavior is resistant to inference from concurrently performing a secondary task (e.g., Logan [18]). The dual-task cost on *general skill learning* (i.e., on the increasing speed due to practice independently from the sequence structure) tends to decrease after mastering the task [19–23]). Nevertheless, no study has directly compared the accessibility of well-acquired probabilistic sequence knowledge (learned without a secondary task) between single and dual-task testing conditions.

Most of the previous experiments testing the effects of dual-tasking on initial learning used fixed (deterministic) sequence learning tasks with first- or second-order adjacent (consecutive) dependencies, i.e., where information on the *n*-1 trial or *n*-1 and *n*-2 together predict the events on trial *n*. The learning of such dependencies might be less implicit than the learning of probabilistic sequences with non-adjacent, higher-order dependencies [15, 17, 24, 25], where the probability of events depends solely on the features of the *n* -2 or earlier trials [26]. (Note that this latter type of learning is often referred to as statistical learning as well due to the acquisition of probabilistic dependencies [25, 27, 28]. However, following with previous dual-task studies, we will use the terms probabilistic sequence learning/knowledge when referring to this type of learning in the remainder of the paper.) The acquisition of such probabilistic non-adjacent dependencies mimics learning in a noisy environment, similarly to how we learn in real life [29], and seems to be crucial for many human skills, including language learning [4, 30, 31]. Therefore, it might not be surprising that probabilistic learning results in robust knowledge [25], and learning likely remains intact when a concurrent secondary task is performed in the initial learning phase [12, 32]. However, others claim detrimental effects [10]. An early study by Schvaneveldt and Gomez [33] found that probabilistic information learned without a secondary task cannot be transferred to a dual-task condition. On the contrary, transfer from dual-task learning to single-task performance did occur, and the authors concluded that the impaired performance was due to problems in the expression of knowledge, and not to the impaired learning itself.

**Fig 1:**
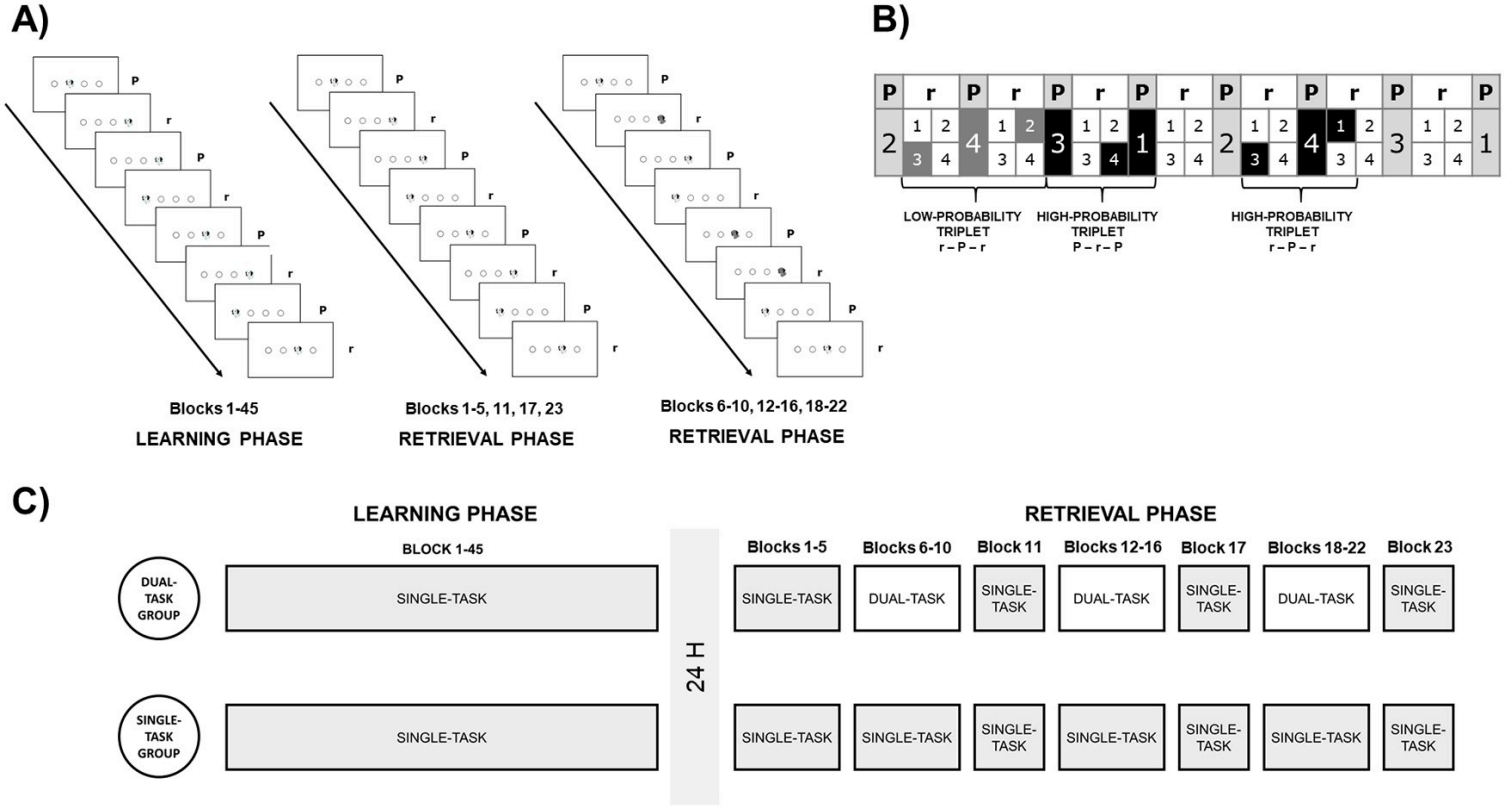
Structure of the ASRT and the experiment. (A) The target stimulus appeared in one of the four possible positions. In the learning phase, only black-and-white stimuli appeared. During the retrieval phase, sometimes yellow stimuli appeared on the screen, which the participants in the dual-task group had to count, while the participants in the single-task group were told to ignore them. (B) High- and low-probability triplets. High-probability triplets (three consecutive elements) can be formed by two patterns (P) and one random element (r) or occasionally, by two random and one pattern elements. (C) Experimental design. In the learning phase, both groups of participants practiced single-task ASRT. After 24 hours, all participants completed five more blocks of single-task ASRT. Then the single-task group completed 18 blocks of single-task ASRT, whereas the dual-task group completed 15 blocks of dual-task ASRT and three blocks of single-task ASRT (one block after every five dual-task blocks).

In the present study, we aimed to investigate the effects of a concurrent secondary task on retrieving implicit probabilistic sequence knowledge with non-adjacent dependencies. So far, studies have investigated the effect of single-task practice on the immediate retrieval of sequence knowledge. However, it is still unclear whether a newly introduced secondary task would disrupt the retrieval of well-acquired non-adjacent probabilities, although it resembles how we pursue dual-task situations in everyday life. Thus, we investigated the effects of a secondary task on the retrieval of well-learned probabilistic sequential knowledge after extended practice, followed by a 24-hour offline period (Fig 1). We trained participants on a probabilistic sequence learning task in a single-task condition. After a 24-hour offline period, participants were tested again; however, at this stage, they performed the task with or without a stimulus-counting secondary task. We chose a stimulus-counting task as the secondary task because most previous studies used similar paradigms [6–8, 15, 34]. To control for the potential differences due to the difficulties in the expression of knowledge [12, 35], we inserted single-task blocks into the retrieval phase of the dual-task group.

Previous diverse findings did not allow us to make clear predictions about the results since they investigated dual-task effects during learning and not during the retention phase. Based on those results, three outcomes were conceivable for our study. First, it was possible that dual-tasking would impair the retrieval of probabilistic sequence knowledge, as the majority of previous studies reported detrimental effects of a secondary task during sequence learning [5–7, 9–11]. Various explanations were offered to account for these results: during dual-tasking, we might disadvantageously integrate the sequenced and non-sequenced information [36], the secondary task might disrupt the organization of the incoming information about regularities [37], or that parallel response selection slows down the learning process [38]. The second possibility was that performing a concurrent task would not influence the retrieval of probabilistic sequence knowledge retrieval. This result would suggest that the retrieval of such knowledge and the successful performance of a stimulus-counting secondary task can be independent from each other—the same way as performance is automatized and resistant to distraction on simple choice-response tasks without sequences to learn [18–23]. The third possibility was that the secondary task would improve the retrieval of the sequence learning task. This outcome would fit well with the hypothesis of competition between control functions and sequence learning abilities [39–42].

## Materials and methods

### Participants

Participants were selected from a pool of eighty-one participants. One participant who did not follow the dual-task instructions in the retrieval phase (see Procedure subsection) was excluded from the pool. We assigned participants into the single-task or dual-task group before the start of the learning phase. As similar initial performance is a crucial criterion in our design, we needed to ensure that the observed effects were not due to differences *before* the dual-task phase. Therefore, we selected a subset of participants from both groups so that the two groups had similar sequence learning performance in the learning phase, which was performed without dual-task (see Procedure subsection, and “*ASRT performance in the learning phase (matching criteria)*” section in S1 Appendix). Data collection and sample selection was performed by different authors. After that, the overall sequence learning performance in the learning phase was compared between the two groups by Bayesian t-test, and sample selection was redone if BF_01_ < 3. Importantly, the random selection was done and finalized prior to analyzing the dual-task phases. The final analyses were carried out on 68 participants: 34 participants in the single-task group (28 males) and 34 in the dual-task group (31 males). Notably, all analyses were also performed on the full sample (*n* = 81), and similar results were observed (see “*Analysis of the full sample*” section in S1 Appendix, as well as S1 Table for additional information on the pool of participants).

The participants were between 18 and 33 years of age (M _age_ = 22.91 years, SD _age_ = 3.48 years). The years of education ranged between 10 and 20 (M _years of education_ = 14.66 years, SD _years of education_ = 2.36 years). Handedness was measured using the Edinburgh Handedness Inventory [43]. The Laterality Quotient (LQ) of the sample varied between −53.85 and 100 (−100 indicates complete left-handedness, 100 indicates complete right-handedness, M _LQ_ = 44.79, SD_LQ_ = 34.22). All participants had normal or corrected to normal vision; none had a history of any neurological and/or psychiatric disorder or reported taking any psychoactive medication at the time of the experiment. They performed in the normal range on the Counting Span task (Range _Counting Span_ = 2.33–6, M _Counting Span_ = 3.81, SD _Counting Span_ = .89) and on the Digit Span task (Range _Counting Span_ = 5–9, M _Counting Span_ = 6.28, SD _Counting Span_ = 1.13). The two groups did not differ in any of the demographic and cognitive characteristics (Table 1). All participants provided written informed consent. Subjects were undergraduate students who participated in the experiment in partial fulfillment of course requirements. Data collection took place in a quiet laboratory room (all measurements in the same room). The study was conducted in accordance with the Declaration of Helsinki and was approved by the Research Ethics Committee of the Eötvös Loránd University, Budapest, Hungary (Ref. no.: 2016/332).

**Table 1:** Comparison of the two groups on age, years of education, handedness, working memory and short term memory performance.

	*Dual-task group*	*Single-task group*	*Group comparison*
	*M(SD)*	*M(SD)*	*(t-test results)*
Age (years)	23.03 (3.42)	22.79 (3.60)	*t*(66) = -.28, *p* = .78
Education (years)	14.50 (2.27)	14.82 (2.47)	*t*(66) = .56, *p* = .58
Handedness (LQ)	50.86 (35.07)	38.73 (32.80)	*t*(66) = -1.47, *p* = .15
Counting Span score	3.92 (0.96)	3.69 (0.81)	*t*(66) = -1.05, *p* = .30
Digit Span score	6.32 (1.15)	6.24 (1.13)	*t*(66) = -.32, *p* = .75

### Required sample size estimation

To ensure that our experiment had enough power, we ran a power analysis with G*Power 3.1.9.7 [44]. No previously published result was available with a similar design (looking for group differences only after a 24-hour offline period) using the Alternating Serial Reaction Time (ASRT) task. Therefore, we calculated the required sample size based on previously published research that used the ASRT task and detected group differences between two independent groups [39, 41, 45, 46]. In these studies, the effect sizes for these comparisons ranged from a η_*p*_^2^ effect size of .12 to .35 (M = .21). Therefore, we estimated a η_*p*_^2^ effect size of .21. With an alpha level of .05 and the desired power level of .90, a sample size of at least 46 participants would be required for detecting group differences. We also verified the number of participants needed to detect group differences in sequence learning for the lowest expected effect size (.12) with a power of .80. This power analysis revealed that 60 participants would be sufficient to obtain significant group differences. These analyses confirmed that our sample of 68 participants was sufficiently large to detect group differences in sequence learning even with the lowest effect size expected based on previous studies.

### Alternating Serial Reaction Time task

We used the ASRT task to test the implicit sequence learning abilities of the participants [47, 48]. Four empty circles were presented continuously in front of a white background arranged horizontally in the middle of a computer screen. A target stimulus (a black and white drawing of a dog’s head) was presented sequentially in one of the four empty circles. Participants were asked to respond with their middle and index fingers of both hands by pressing the button corresponding to the target position on a keyboard with four marked keys (Z, C, B, and M on a QWERTY keyboard), each of the four keys corresponding to the circles in a horizontal arrangement (Fig 1A). Participants were instructed to be as fast and as accurate as possible.

The serial order of the four possible locations (coded as 1, 2, 3, and 4) in which target stimuli could appear was determined by an eight-element probabilistic sequence. In this sequence, every first element appeared in the same order as the task progressed. The second elements’ positions were randomly chosen out of the four possible locations (e.g., 2r4r3r1r; where r indicates a random position). Thus, some combinations of three consecutive trials (*triplets*) occur with a higher probability than other combinations. For example, 2X**4**, 4X**3**, 3X**1**, and 1X**2** (where ‘‘X” indicates any possible middle element of the triplet) would occur with high probability because the third element (bold numbers) could be derived from the sequence (or occasionally could be a random element as well). In contrast, 1X**3** or 4X**2** would occur with less probability because these triplets cannot be formed from two sequence elements and one random element. Therefore, the third element of a high-probability triplet is more predictable from the first element when compared to a low-probability triplet.

There were 64 possible triplets in the task altogether. Sixteen of them were high-probability triplets, each occurring in approximately 4% of the trials, five times more often than each of the remaining 48 low-probability triplets (0.8%). Overall, high-probability triplets occur with approximately 62.5% probability, while low-probability triplets only occur with 37.5% probability (Fig 1B). As the participants practice the ASRT task, their responses become faster and more accurate to the high-probability triplets compared to the low-probability triplets, revealing sequence-specific learning [25, 47, 49, 50]. Six different sequences were used across subjects, but the sequence for a given subject was identical throughout the entire experiment.

The ASRT task was completed in blocks, and each block contained 85 button presses (five random elements at the beginning of the block; then, the eight-element alternating sequence was repeated ten times). At the beginning of each block, four empty circles were presented horizontally on the screen for 1000 ms, and then the first target stimulus appeared. The target stimulus remained on the screen until the first correct response. The participants received feedback about their performance on the screen (average RT and accuracy) and could rest a little between blocks. After five blocks, a longer (5 min) mandatory pause was inserted.

We defined each trial as the third element of a high- or low-probability triplet. Trills (e.g., 1-2-1) and repetitions (e.g., 1-1-1) were eliminated from the analysis because participants may show pre-existing response tendencies for these types of triplets [28, 49, 51, 52]. The first button presses were also excluded from the analysis (first five random button presses, and the 6^th^ and 7^th^, as they cannot be evaluated as the third element of a triplet). To facilitate data analysis and increase the signal-to-noise ratio, every five blocks were collapsed into a larger analysis unit.

### Procedure

The experiment consisted of two sessions (Fig 1C). In the learning phase, participants completed 45 blocks of ASRT (45 blocks divided into 9 units of analysis: blocks 1–5, blocks 6–10, blocks 11–15, blocks 16–20, blocks 21–25, blocks 26–30, blocks 31–35, blocks 36–40, and blocks 41–45), which is long enough to acquire stable statistical knowledge [53].

The retrieval phase was held 24 hours after the learning phase. Participants completed 23 blocks of ASRT with the same sequence that they previously learned (20 blocks divided into four units of analysis (epochs): blocks 1–5, blocks 6–10, blocks 12–16, blocks 18–22), and three separate blocks intended as control blocks, without dual-task condition (block 11, block 17, and block 23). In blocks 1–5, the instructions were completely the same as in the previous day. This phase was included to strengthen the acquired probabilistic sequence knowledge and to ensure that the two groups consolidated the knowledge to a similar level (see “*ASRT performance in the learning phase (matching criteria)”* section in S1 Appendix). However, in blocks 6–10, blocks 12–16, and blocks 18–22, a random number of stimuli (40–45 out of the 85 appearing stimuli in one block) was colored in yellow. The dual-task group was instructed to count the number of yellow dogs throughout these blocks. After completing the given block, the participants had to type the number of yellow dogs they had counted. The yellow-colored stimuli also appeared for the single-task group. However, they were instructed to carry on with the task without paying particular attention to the differently colored stimuli.

Performance in the secondary task was evaluated by calculating the difference from the correct number of yellow stimuli divided by the total number of yellow stimuli for each unit of five blocks (thus, resulting in a percentage score of correctly counted yellow stimuli relative to the total number of yellow stimuli). If the participant’s overall difference score was over 15%, the participant was considered not to follow the instructions and was excluded from the analysis (one participant).

Two control blocks were inserted between the three dual-task phases (block 11 and block 17), and another at the end of the session (block 23). In these blocks, there were no yellow stimuli for the dual-task, or for the single task group. The dual-task group was told to continue the task without counting any stimulus. At the beginning of the next dual-task phase, they were instructed again to count the yellow stimuli.

Following the ASRT task, the Inclusion-Exclusion task was administered to check whether the participants developed conscious knowledge about the learned probabilistic regularities (see “*Inclusion-Exclusion*” section in S1 Appendix).

### Statistical analysis

To evaluate the performance in the ASRT task, we calculated the median reaction times (RTs) separately for the high- and low-probability triplets in every five blocks of the learning phase (45 blocks: blocks 1–5, blocks 6–10, blocks 11–15, blocks 16–20, blocks 21–25, blocks 26–30, blocks 31–35, blocks 36–40, and blocks 41–45), the retrieval phase (20 blocks: blocks 1–5, blocks 6–10, blocks 12–16, blocks 18–22), and in the three control blocks of the retrieval phase (3 blocks: block 11, block 17, and block 23). Only correct responses were included in the RT analysis. We focused on the analysis of RTs, as previous similar ASRT studies have observed ceiling effects in accuracy [28].

We calculated *learning scores* by subtracting the RTs for high-probability triplets from the RTs for low-probability triplets. To test the effects of the secondary task, we compared (1) the performance *while* the dual-task group performed the dual-task (test) blocks, and (2) the performance *between* the dual-task phases (control blocks). Moreover, we directly compared the *learning scores* between the test and control blocks in the two groups to test if the probabilistic sequence knowledge was different during the two phases.

As dual-tasking caused major differences in median RTs between the two groups in the dual-task blocks, we wanted to ensure that the probabilistic sequence knowledge results were not due to the changes in the overall speed (i.e., because of the effect of the dual-task on *general skill learning*). To this end, we performed an additional analysis of the data with standardized scores. The standardized RT scores were calculated by dividing the learning scores by the *average* RTs of the given unit of five blocks for each participant and each unit of five blocks. Moreover, we analyzed how the overall RTs were affected by the dual-task by comparing the overall RTs between the test blocks and control blocks and between the two groups.

For all ANOVAs, the Greenhouse-Geisser epsilon (ε) correction was used if necessary. Corrected *df* values and corrected *p* values are reported (if applicable) along with partial eta-squared (η_*p*_^2^) as the measure of effect size. LSD (Least Significant Difference) tests were used for pairwise comparisons. The alpha level was set at .05 for all analyses.

In addition to the frequentist analyses (NHST–null hypothesis significance testing), we conducted Bayesian independent samples t-tests on the relevant comparisons. Moreover, we conducted Bayesian ANOVA on the learning scores to quantify each factor’s contribution to the results. Here, we present Bayesian Model Averaging, and we report the inverted BF_inclusion_ values (1/BF_inclusion_ = BF_exclusion_). These values indicate whether the exclusion of the given factor from the model is supported by our data (values > 1 indicate evidence in favour of exclusion, while values < 1 indicate evidence for inclusion). We decided to include both NHST and Bayesian analyses for two reasons: 1) to report the result in a more conventional way (NHST) and 2) to gain statistical evidence for potential null-results [54]. Frequentist analyses were carried out using IBM SPSS Statistics 25 and Bayesian analyses using JASP [55].

## Results

### Did the retrieval of the sequence knowledge differ between groups in the test blocks?

Probabilistic sequence knowledge was retained, as suggested by shorter RTs for high vs. low-probability triplets (see details in “*Comparison of RTs for high- and low-probability triplets*” section of S1 Appendix and S1 Fig). To compare the probabilistic sequence knowledge of the two groups in the test blocks (when the dual-task group performed the secondary task), we performed a mixed-design ANOVA on the learning scores with the within-subject factor of Block (retrieval phase blocks 6–10 vs. retrieval phase blocks 12–16 vs. retrieval phase blocks 18–22) and the between-subject factor of Group (dual-task group vs. single-task group).

**Fig 2:**
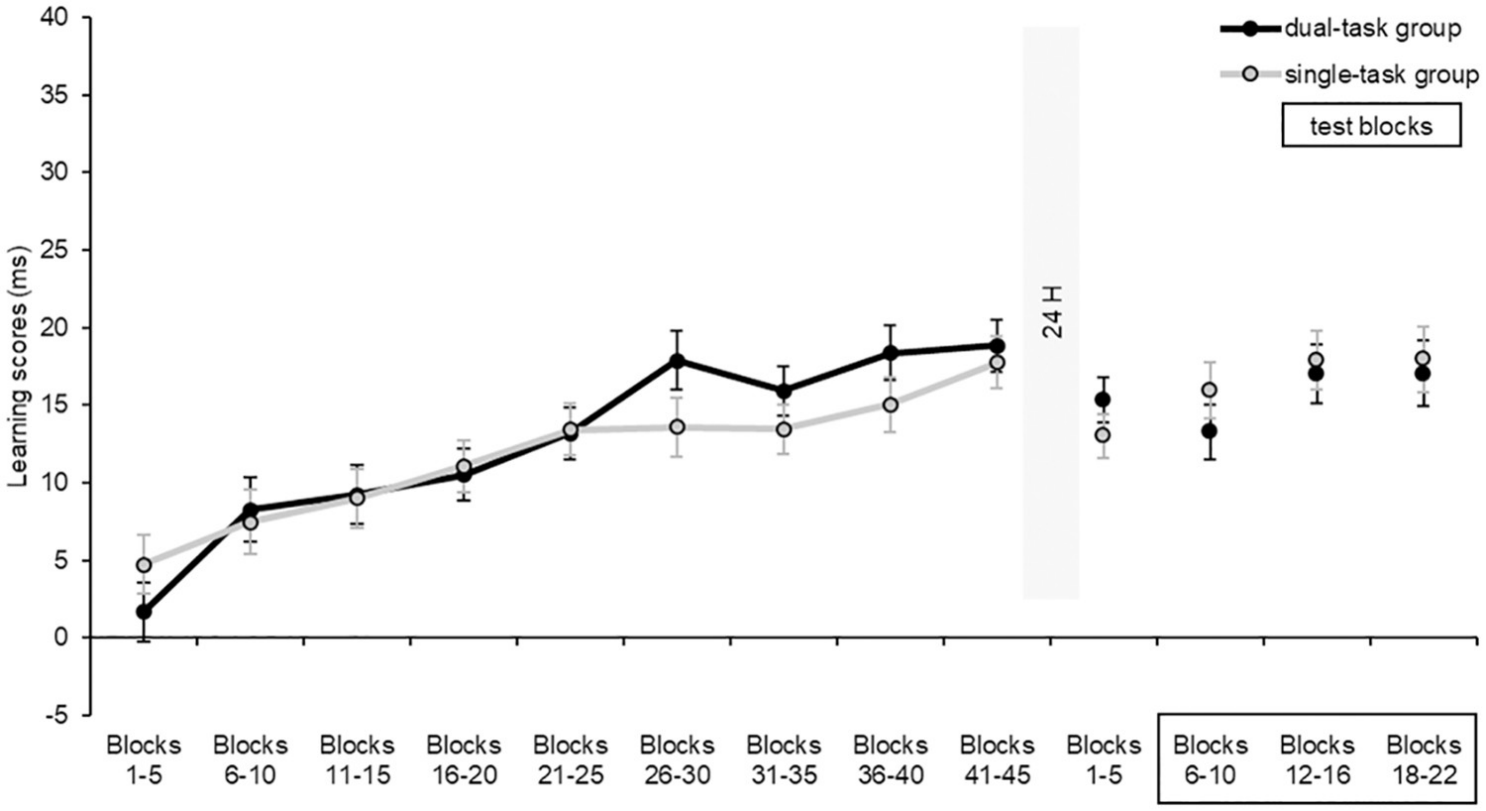
Learning scores of the two groups during the learning and the retrieval phase. The vertical axis represents the learning scores (the difference between RTs for high- and low-probability triplets) in ms, and the horizontal axis represents the nine units of five blocks of the learning phase (block 1–45) and the four units of five blocks of the retrieval phase (blocks 1–5, blocks 6–10, blocks 12–16, blocks 18–22). The black line represents the learning scores of the dual-task group, and the grey line the learning scores of the single-task group. The squared block units represent the test blocks, i.e., when the secondary task was also performed by the dual-task group. The error bars represent the standard error of the mean. At the beginning of the retrieval phase, stable statistical knowledge was detected in blocks 1–5. The statistical knowledge remained stable in the later parts of the retrieval phase, even during the test blocks, and no difference was found between groups in terms of learning scores. We found similar results with standardized scores.

The ANOVA on the learning scores did not reveal a significant main effect of Block, *F*(2, 132) = 2.26, *p* = .11, η_*p*_^2^ = .03, BF_exclusion_ = 3.93, indicating that the degree of sequence-specific learning did not change significantly throughout the test blocks. Importantly, the main effect of Group did not reach significance, *F*(1, 66) = 0.52, *p* = .47, η_*p*_^2^ = .01, BF_exclusion_ = 4.87, revealing that no significant difference was observable between groups in the degree of sequence-specific knowledge. This lack of difference did not change throughout the blocks, as revealed by a non-significant Block × Group interaction, *F*(2, 132) = 0.21, *p* = .81, η_*p*_^2^ = .003, BF_exclusion_ = 37.97 (Fig 2).

### Did the sequence knowledge of the two groups differ in the test blocks measured by the standardized scores?

The Block × Group ANOVA of the *standardized learning scores* did not reveal a significant main effect of Block, *F*(2, 132) = 1.39, *p* = .25, η_*p*_^2^ = .02, BF_exclusion_ = 8.33, suggesting that the learning scores did not change significantly during the test blocks. Importantly, consistent with the results without standardization, no group difference was found in the degree of sequence-specific knowledge (main effect of Group: *F*(1, 66) = 1.33, *p* = .25, η_*p*_^2^ = .02, BF_exclusion_ = 3.70). This lack of significant difference remained stable throughout the test blocks, as revealed by a non-significant Block × Group interaction, *F*(2, 132) = .02, *p* = .98, η_*p*_^2^ *<* .001, BF_exclusion_ = 64.84.

### Did the sequence knowledge of the two groups differ in the control blocks?

**Fig 3:**
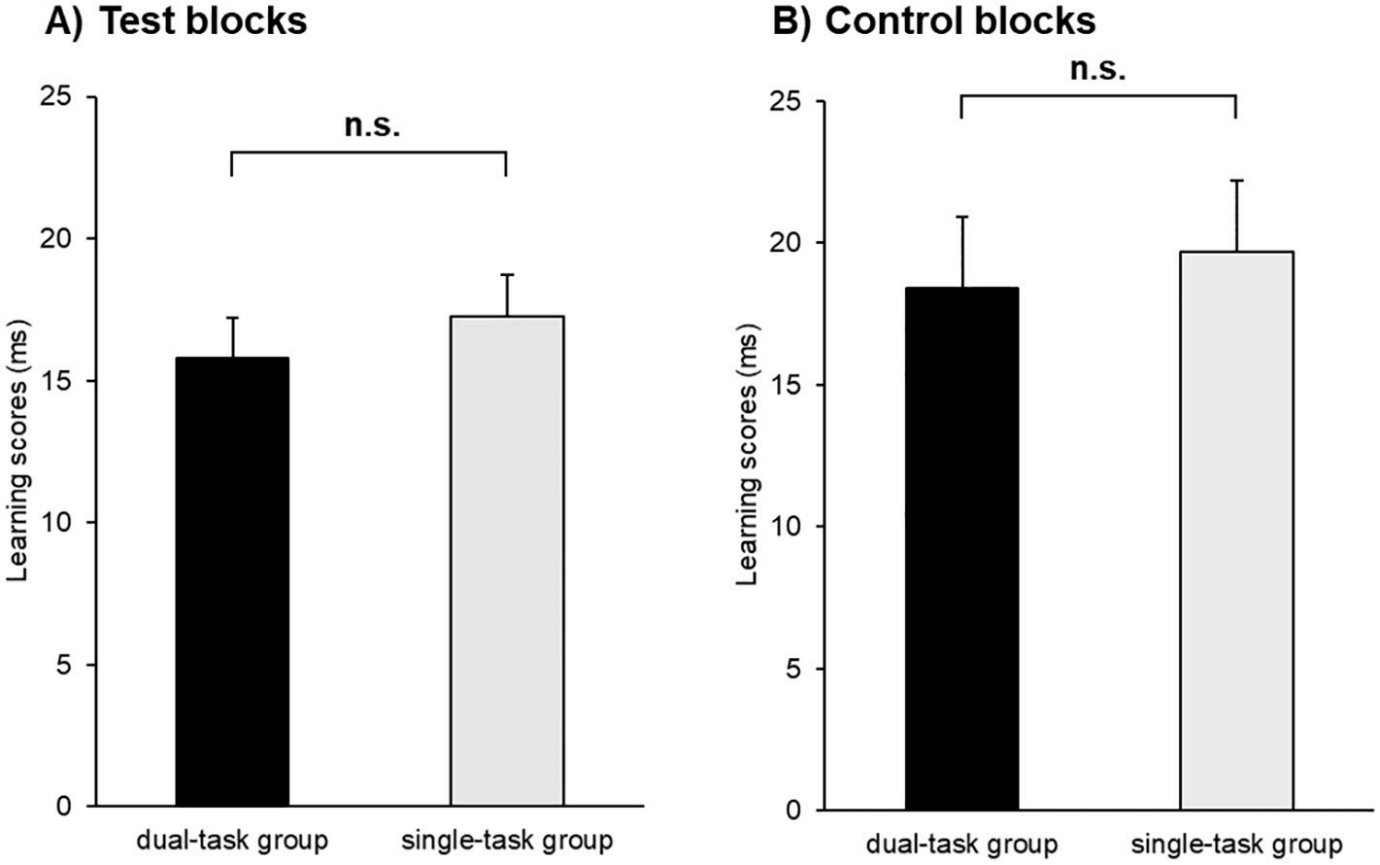
The learning scores of (A) the test blocks and (B) control blocks of the retrieval phase. The horizontal axes represent the two groups. The vertical axes indicate the learning scores (RTs for the low-probability triplets *minus* RTs for the high-probability triplets, the blocks collapsed together). The error bars signal the standard error of the mean. The learning scores of the two groups did not differ in the test blocks or the control blocks. We found similar results with standardized scores. *n*.*s*.: *p >* .*05*.

We checked if the groups performed differently in the control blocks (block 11, block 17, and block 23). As one block contains only 85 button presses, we averaged over the three blocks to gain more statistical power. The independent sample t-test revealed a lack of difference between groups in the degree of probabilistic sequence knowledge, *t*(66) = -0.36, *p* = .72, BF_01_ = 3.80 (Fig 3).

### How did the learning scores of the test blocks and the control blocks compare?

We examined whether the learning scores of the test blocks and the control blocks differed and whether the degree of probabilistic sequence knowledge was similar between the two groups. The Block Type × Group ANOVA on the learning scores did not reveal a significant main effect of Block Type, *F*(1, 66) = 1.87, *p* = .18, η_*p*_^2^ = .03, BF_exclusion_ = 3.15, suggesting a lack of significant difference in the degree of probabilistic sequence knowledge between the test and control blocks (i.e., between the periods where the stimulus stream contained colored stimuli). The main effect of Group did not reach significance, *F*(1, 66) = 0.38, *p* = .54, η_*p*_^2^ = .006, BF_exclusion_ = 5.20, indicating that the two groups performed similarly on the second session. More importantly, there was no significant difference between the two groups in how the learning scores developed between the two types of blocks, as suggested by the non-significant interaction of the Block Type and Group factors, *F*(1,66) = 0.004, *p* = .95, η_*p*_^2^ < .001, BF_exclusion_ = 15.18.

### Did the dual-tasking change overall RTs?

**Fig 4:**
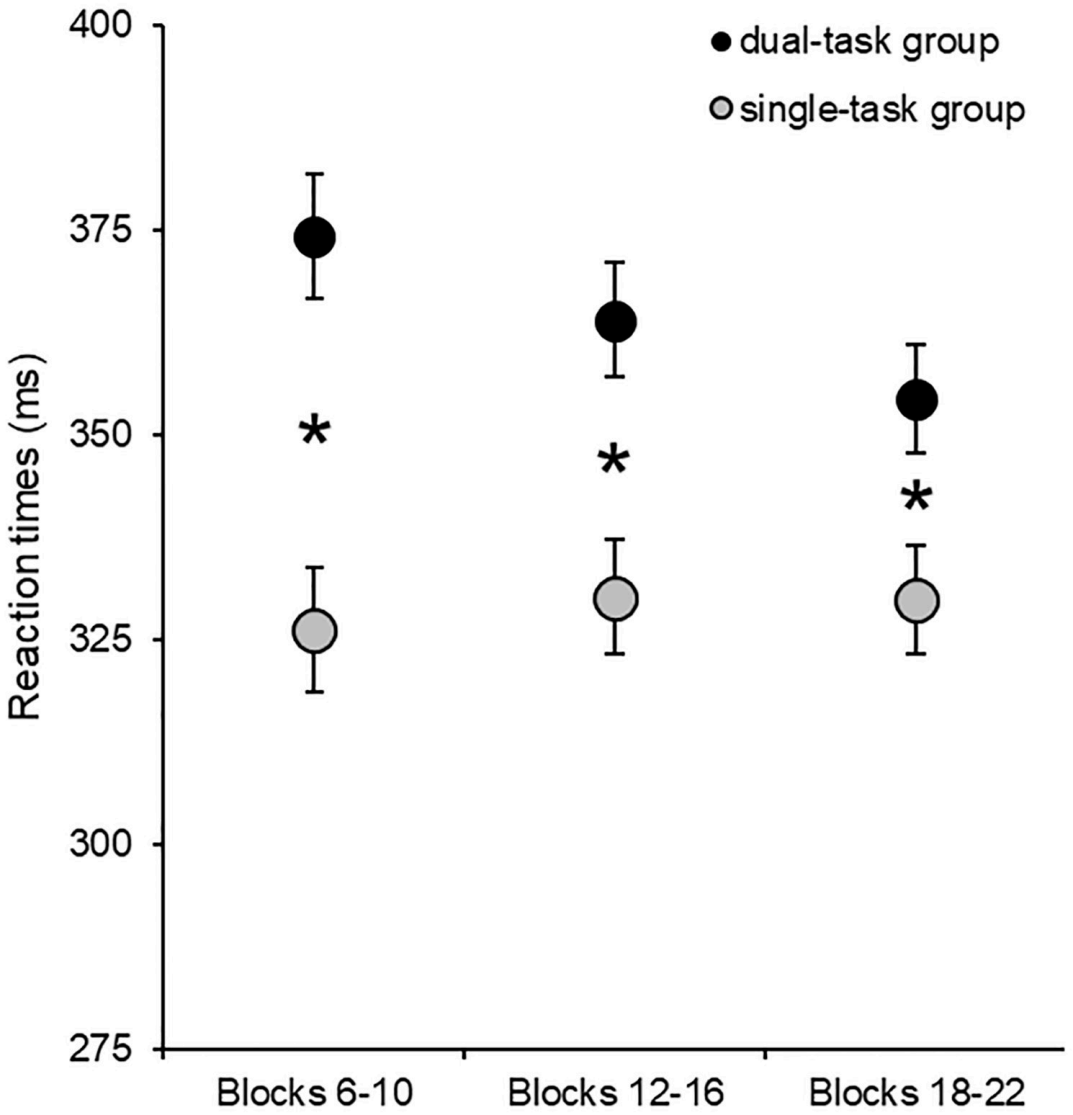
Overall RTs in the test blocks. The horizontal axis represents the three units of test blocks, and the vertical axis the average RTs. The error bars signal the standard error of the mean. Participants were faster in the test blocks in the single-task group, revealing that the dual-task affected the average RTs. *: *p <* .*05*.

We tested whether the dual-task affected overall RTs independently of the triplet types. We ran a mixed-design ANOVA on the average RTs with the within subject-factor of Block (retrieval phase blocks 6–10 vs. retrieval phase blocks 12–16 vs. retrieval phase blocks 18–22) and the between-subject factor of Group (dual-task group vs. single-task group). The ANOVA revealed a significant main effect of Block, *F*(1.67, 109.86) = 11.00, *p* < .001, η_*p*_^2^ = .14, BF_exclusion_ = 6.58e-9. This result indicates that overall RTs became shorter as the task progressed (general skill learning). As expected, the main effect of Group was also significant, *F*(1,66) = 13.11, *p* < .001, η_*p*_^2^ = .17, BF_exclusion_ = 1.05e-8. This result indicates that overall RTs were larger in the dual-task group than in the single-task group (Fig 4). The Block × Group interaction was also significant, *F*(1.67, 109.86) = 23.40, *p* < .001, η_*p*_^2^ = .26, BF_exclusion_ = 5.07e-8, indicating that the acceleration of RTs was only detectable in the dual-task group.

We also examined if the overall RTs differed in the control blocks. We repeated the above analysis with the control blocks instead of the test blocks. The ANOVA did not reveal a significant main effect of Control Block, *F*(2, 132) = 0.21, *p* = .82, η_*p*_^2^ = .003, BF_exclusion_ = 22.88, indicating similar RTs in the three control blocks. The main effect of Group was also non-significant, *F*(1, 66) = 2.69, *p* = .11, η_*p*_^2^ = .04, BF_exclusion_ = 1.37, indicating no difference in overall RTs between groups. The Block × Group interaction was also non-significant, *F*(2, 132) = 0.08, *p* = .92, η_*p*_^2^ = .001, BF_exclusion_ = 95.40, suggesting no difference in the (lack) of change in overall RTs between the groups.

**Fig 5:**
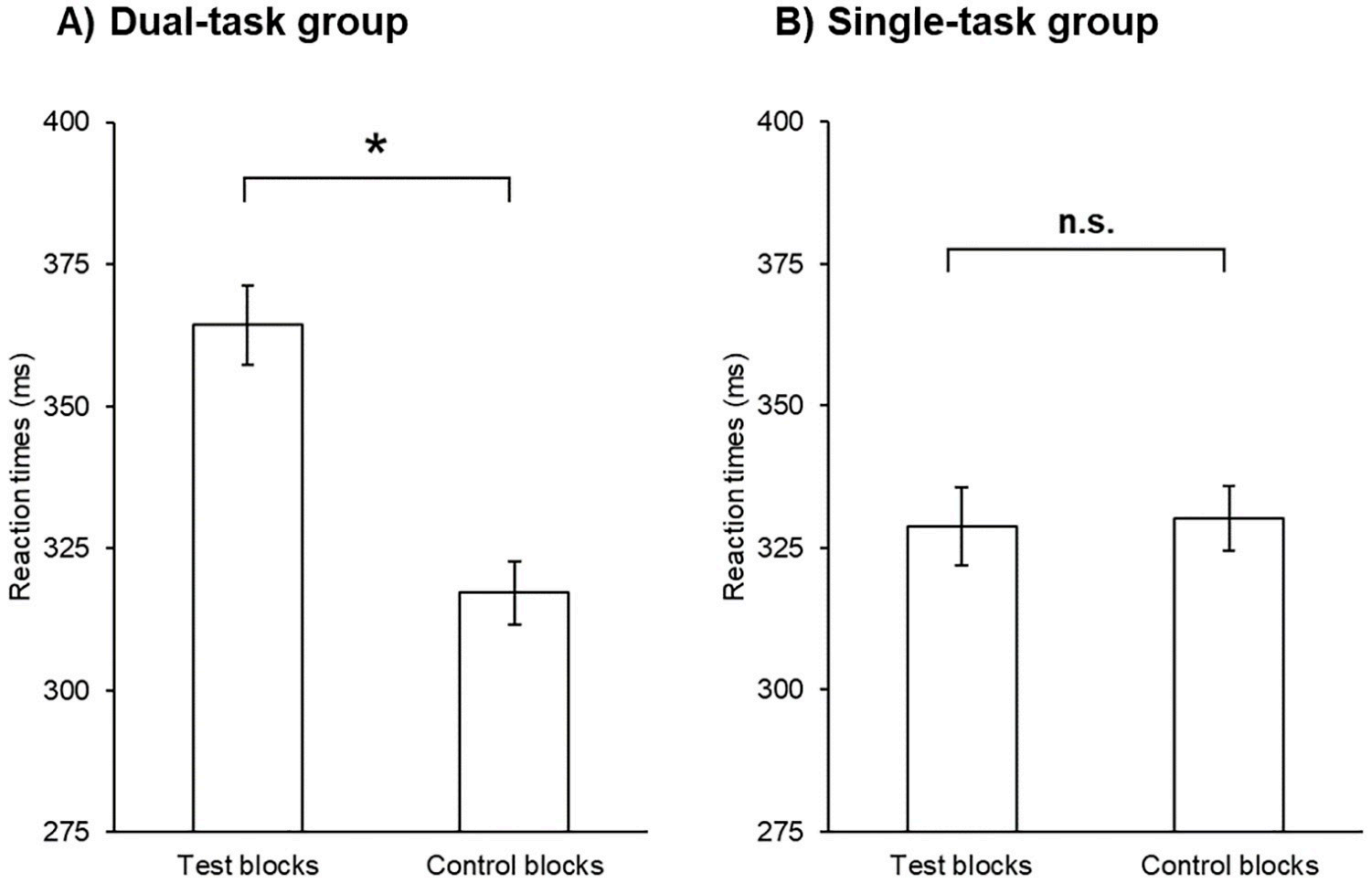
Overall RTs between the test and control blocks in (A) the dual-task group and (B) single-task group. The horizontal axis represents the two types of blocks, and the vertical axis the average RTs. The error bars signal the standard error of the mean. Participants in the dual-task group were faster in the control blocks compared to the test blocks. This pattern was not confirmed in the single-task group, revealing that the dual-task affected the average RTs. n.s.: *p >* .05; *: *p <* .*05*.

Next, we also compared the overall RTs between the two types of blocks and the two groups. To this end, we used the overall RTs of the three units of test blocks and the three control blocks. The Block Type × Group ANOVA revealed a main effect of Block Type, *F*(1, 66) = 109.29, *p* < .001, η_*p*_^2^ = .62, BF_exclusion_ = 0.001, indicating that participants were generally slower in the dual-task phase. The main effect of Group was non-significant, *F*(1, 66) = 1.70, *p* = .20, η_*p*_^2^ = .03, BF_exclusion_ = 1.21e-13, indicating an overall similar performance between the two groups. However, the Block Type × Group interaction was significant, *F*(1, 66) = 123.30, *p* < .001, η_*p*_^2^ = .65, BF_exclusion_ = 3.38e-14. In the dual-task group, slower RTs were detected in the test blocks compared to the control blocks (*p* < .001), while no difference was found in the single-task group (*p* = .65) (Fig 5).

## Discussion

Here, we investigated the effect of a secondary task on the retrieval of well-established implicit sequence knowledge of probabilistic non-adjacent dependencies. Participants practiced a probabilistic sequence learning task with non-adjacent second-order dependencies throughout 45 blocks. After a 24-hour offline period and a reactivation phase, participants were tested with or without a concurrent stimulus-counting task. Participants examined under dual-task conditions retrieved their probabilistic sequence knowledge to the same level as participants with only single-task testing conditions. This similarity persisted during blocks where both groups retrieved their knowledge under single-task conditions. The results remained the same, even when the differences in overall RTs between groups were controlled. Bayesian statistical methods also confirmed the lack of difference between groups in implicit sequence knowledge. Moreover, the lack of difference between groups remained when all participants were included in the analysis (see “*Analysis of the full sample*” section in S1 Appendix). The dual-task affected overall RTs demonstrating that the dual-task manipulation was efficient, although the retrieval of sequence-specific knowledge was intact. Our results went beyond the previous literature by showing that well-established knowledge of probabilistic non-adjacent dependencies can be resistant to a concurrent secondary task.

Our study could have resulted in three possible outcomes: impaired, intact, or improved retrieval of the learned probabilities under dual-task conditions compared to single-task retrieval. Expecting impaired performance can seem reasonable at first, as the majority of previous studies reported deteriorating effects of a secondary counting task on sequence learning [5–7, 9–11]. This detrimental effect on learning was explained by numerous theories such as the suppression hypothesis [15, 34], task integration [36], organizational hypothesis [37], or the response selection hypothesis [38]. However, other studies revealed intact or even improved learning, especially in the case of complex probabilistic regularities [4, 7, 12, 33].

An essential difference between previous studies and ours is that we introduced the secondary task after a considerable amount of practice on the primary task. The participants completed more than 4000 trials on the primary task before introducing the dual-task condition. In comparison, the practice on the primary task ranged from zero to a few hundred trials in most of the previous studies. With this modification, we did not find evidence for impaired proficiency in retrieving the learned information under dual-task conditions. Contrary to our results, an early study by Schvaneveldt and Gomez [33] found that after initial single-task learning, participants could not apply the knowledge of probabilities to a dual-task condition; however, it was not the case when the initial learning occurred under dual-tasking. Their study tested the transfer to a dual-task condition within one session (with less practice and shorter retention period). On the contrary, we implemented a longer practice period, a 24-hour offline period, and a reactivation period to ensure that the sequence is well-learned before the retrieval. This extensive practice before introducing the dual-task might have led to a takeover of the automatic systems on sequence knowledge [56]. Then, in the dual-task condition, the presentation of yellow stimuli might have interfered with the visual coordinates of sequence representations. Thus, it might have slowed down the more explicit/controlled responses ruling general skills, while the difference in responses to the sequence elements with different probabilities persisted. Sequence learning might thus be supported by an encapsulated system, protecting it from the dual-task condition [57], whereas general skill might rely on a more multimodal system, integrating information from several modalities, and could thus be affected by the insertion of random items (yellow stimuli) which are not related to the primary task. Nevertheless, our results suggest that acquiring the probabilistic sequence knowledge to a great extent might help maintain an adequate level of retrieval during a subsequent dual-task condition.

Apart from the secondary task’s potentially disruptive effect, another possible outcome of the study was that the concurrent task would leave access to the probabilistic sequence knowledge intact. This would indicate that the processes behind the retrieval of such knowledge and the stimulus-counting secondary task are independent from each other, similarly to how performance becomes automatized and resistant to dual-tasking on simple choice-response tasks without underlying sequence information [18–23]. When skill-related memory representations are formed, they cease to rely on the same resources.

Our main results are in line with this prospect: the degree of sequence knowledge remained the same compared to the single-task retrieval (please note that in our study, the sequence knowledge became automatized but not the perceptual-motor improvement, see below). Moreover, the lack of differences persisted even after controlling for the differences in baseline reaction times. The fact that the probabilistic sequence knowledge of the dual-task group was comparable to that of the single-task group both in the dual-task blocks (performance) and in the intermittent control blocks (competence) indicates that the secondary task did not affect the performance or the competence of the primary task [35, 58].

These results are consistent with previous research that found intact implicit sequence knowledge after practicing the primary task in single-task conditions [15, 16]. However, in these studies, the presentation of the dual-task blocks immediately followed the few single-task learning blocks limiting its generalizability for conditions where the retrieval of the acquired knowledge under dual-task occurs after a longer offline period. Our results thus extend the knowledge on the effect of dual-tasking on sequence learning/retrieval by providing evidence for three additional aspects. First, the retrieval of probabilistic sequence knowledge remains resistant to a concurrent task even after a 24-hour offline period, which underscores the robust nature of probabilistic sequence learning [25, 59]. Second, the retrieval of implicit probabilistic representations remains intact after extended practice (see “*ASRT performance in the learning phase (matching criteria)*” in S1 Appendix). Third, neither the competence nor the performance of a well-established knowledge of probabilistic dependencies can be disrupted by a secondary task.

The third potential outcome of the study was that the secondary task would improve the retrieval of the memory representations of the primary probabilistic sequence learning task. This possibility would fit well with the competition theory [60, 61], which posits an antagonistic relationship between basal ganglia vs. prefrontal/medial temporal lobe-dependent learning and memory processes, as well as neurocognitive functions supporting them, such as cognitive control or executive functions. Accordingly, several studies showed negative correlations between control functions and probabilistic sequence learning [39–42]. Moreover, the non-invasive inhibition of prefrontal cortical areas has been shown to lead to increased sequence learning abilities [53, 62], providing causal evidence for the competition theory. Based on these results, one could expect that a demanding secondary task would facilitate access to sequence representations. However, we did not find improved performance in the dual-task condition. A possible explanation is that the competition theory is not applicable in dual-task situations. However, a more plausible explanation is related to the specificity and characteristics of the secondary task, as our secondary and primary task shared common input modality [63]. Although the prolongation of RTs during the secondary task confirms that our stimulus-counting task is distracting and dual-task costs emerge in overall RTs, this task may not engage mechanisms that trigger competitive interactions resulting in better performance in the retrieval of probabilistic sequence knowledge [6]. The exploration of which secondary tasks (if any) might be advantageous for the retrieval of such knowledge deserves future investigations.

Beyond the interpretation of the obtained results in the current theoretical frameworks, methodological aspects can also account for the results. The ASRT task allows us to disentangle general skill-related processes and sequence-specific knowledge. The former was not taken into account by many previous studies, hindering their ability to unveil the underlying mechanism behind dual-task effects. In our study, the change of the overall RTs shows general skill-related processes such as perceptual-motor coordination and adaptation to the experimental situation. At the same time, sequence-specific knowledge is considered to be the emergence of a difference between high and low-probability triplets (often referred to as statistical learning as well due to the acquisition of probabilistic dependencies in the practiced sequence, [25, 27, 28]). It is important to note that the increased overall RTs during the retrieval phase under dual-task conditions did not reveal impaired probabilistic sequence knowledge: they indicate altered general skill retrieval on the primary task due to the dual-task constraint. The secondary task slowed down the overall perceptual-motor coordination (as evidenced by the results of the overall RTs), suggesting that in this aspect, the performance was not automatized until this point. We can interpret the change in the overall RTs as dual-tasking extends the time needed to access the acquired knowledge of the probabilistic dependencies. However, this does not mean that the degree of the acquired knowledge to which participants had access has changed.

The sequence knowledge that emerged during the learning phase became robust enough to persist under dual-tasking; thus, we found a dissociation between the two processes. After the normalization of the baseline RTs, the lack of differences in sequence knowledge between the single- and dual-task groups persisted. This result supports the dissection of general skill learning and probabilistic sequence learning in our study. It is crucial for stating that probabilistic sequence knowledge was similar between the groups as general skill and sequence learning were differentiated by previous studies [64]. Previous inconsistencies in the dual-task literature might also originate from differences in the proportion of general skill- and sequence learning-related factors of the used task. Therefore, future studies investigating the process of sequence learning or the retrieval of the acquired sequence knowledge under dual-task conditions could benefit from considering these aspects as potential confounding factors.

Previous studies have tried to determine which characteristics of the secondary tasks are crucial for disrupting the learning process, such as the correlation between the primary and secondary task events or the features of the required response [9]. In our study, we chose a visual secondary task implemented in the stimulus stream of the primary task. It does not break the stimulus-response interval, which has been proposed to cause interference between the tasks or to trigger selective instead of divided attention—such as tone counting tasks might do [12]. However, we do not know if different secondary tasks involving functionally distant cognitive processes affect the retrieval of sequence knowledge to a similar extent. For example, sentence processing was found to impair probabilistic sequence learning, while mathematical and word processing tasks did not have disruptive effects [4]. This result can be explained by the fact that language processing relies on non-adjacent dependencies, similarly to the probabilistic sequence learning task used in the current study. Interestingly, using a serial reaction time task with adjacent dependencies, sequence learning was also boosted when the secondary task involved similar sequence-learning processes as the primary task [6]. Therefore, the set of cognitive processes that can and cannot interfere with the retrieval of sequence information is yet to be empirically established.

In everyday life, we mostly perform a secondary task when the primary task is well-acquired. Despite this fact, to the best of our knowledge, no study had investigated the effect of a secondary task on the retrieval of well-acquired, non-adjacent probabilistic regularities. To fill this gap, we exposed participants to a secondary task after extensive practice on the primary implicit probabilistic sequence learning task. We found an intact retrieval of implicit probabilistic sequence knowledge, providing evidence that non-adjacent probabilistic representations can be robust against dual-tasking even if the general skill learning aspect of the primary task is affected. This result suggests that the representations of non-adjacent probabilistic regularities become more resistant to disruption than the general skill learning and that we can correctly apply the acquired knowledge of probabilistic regularities if we are performing a secondary task concurrently. Our results emphasize the importance of studying the dual-task effect not only during the learning phase but also during memory retrieval to reveal the robustness of the acquired skill.

## Supporting information

S1 Appendix Supplementary materials. (DOCX)

S1 Fig Comparison of RTs for high- and low-probability triplets. (TIF)

S1 Table Demographic characteristics of the full sample. (DOCX)
