# Supplementary material for: Retrieval of a well-established skill is resistant to distraction: Evidence from an implicit probabilistic sequence learning task
Source: PLoS One. 2020 Dec 10;15(12):e0243541. doi: 10.1371/journal.pone.0243541 (PMC7728172; doi:10.1371/journal.pone.0243541)
Supplement: S1 Appendix — (DOCX) [file pone.0243541.s001.docx]

Teodóra Vékony^1,2 ¶^ , Lilla Török^3 ¶^ , Felipe Pedraza^1,4^, Kate Schipper^1^, Claire Pleche^1^, László Tóth^3^, Karolina Janacsek^5,6,7 &^ , Dezso Nemeth^1,6,7 &*^

^1^ Lyon Neuroscience Research Center (CRNL), INSERM, CNRS, Université Claude Bernard Lyon 1, Lyon, France

^2^ Department of Neurology, University of Szeged, Szeged, Hungary

^3^ Department of Psychology and Sport Psychology, University of Physical Education, Budapest, Hungary

^4^ Institute of Psychology, Université Lumière - Lyon 2, Lyon, France

^5^ Centre for Thinking and Learning, Institute for Lifecourse Development, School of Human Sciences, Faculty of Education, Health and Human Sciences, University of Greenwich, London, United Kingdom

^6^ Institute of Psychology, ELTE Eötvös Loránd University, Budapest, Hungary

^7^ Brain, Memory and Language Research Group, Institute of Cognitive Neuroscience and Psychology, Research Centre for Natural Sciences, Budapest, Hungary

# Comparison of RTs for high- and low-probability triplets

## Did the sequence knowledge of the two groups differ in the test blocks?

We tested whether the two groups performed differently in the test blocks (when the dual-task group performed the secondary task) on the two triplet types. A mixed-design ANOVA on the RT scores with the within-subject factors of Triplet (high- vs. low-probability) and Block (retrieval phase blocks 6-10 vs. retrieval phase blocks 12-16 vs. retrieval phase blocks 18-22) and the between-subjects factor of Group (dual-task group vs. single-task group) was performed.

A significant main effect of Block was found, *F*(1.67, 109.86) = 11.00, *p* < .001, η*_p_*^2^ = .14, highlighting that the average RTs accelerated during the retrieval phase. The significant main effect of Triplet, *F*(1, 66) = 254.19, *p* < .001, η*_p_*^2^ = .79, showed that probabilistic sequence knowledge was still detectable during the test blocks. The Block × Triplet interaction was not significant, *F*(2, 132) = 2.26, *p* = .11, η*_p_*^2^ = .03, indicating that the degree of probabilistic sequence learning did not change significantly throughout the test blocks.

The main effect of Group was significant, *F*(1, 66) = 13.11, *p* < .001, η*_p_*^2^ = .17, revealing that the average RTs were higher in the dual-task group than in the single-task group. This suggests that completing the secondary task during the ASRT gave rise to a general slowing down in the task. The Block × Group interaction was significant, *F*(2, 132) = 23.40, *p* < .001, η*_p_*^2^ = .26, indicating that the general RT decrease was only detectable in the dual-task group (all blocks differed from each other, all *p*s < .001); the single task group did not show acceleration following the learning phase (neither block was different from the others: *p* > .09). Importantly, the Triplet × Group interaction did not reach significance, *F*(1, 66) = 0.52, *p* = .47, η*_p_*^2^ = .01, revealing that there was no statistically significant difference between groups in terms of the degree of probabilistic sequence knowledge. This lack of difference did not change throughout the blocks, as revealed by a non-significant Block × Triplet × Group interaction, *F*(2, 132) = 0.21, *p* = .81, η*_p_*^2^ = .003.

## Did the sequence knowledge of the two groups differ in the control blocks?

We checked if the groups performed differently in the control blocks that were administered between the test blocks (block 11, block 17, and block 23). As one block contains only 85 button presses, we averaged over the three blocks to gain more statistical power. The Triplet × Group ANOVA revealed a main effect of Triplet, *F*(1, 66) = 114.51, *p* < .001, η*_p_*^2^ = .63, revealing stable probabilistic sequence knowledge. The main effect of Group was not significant, *F*(1, 66) = 2.69, *p* = .11, η*_p_*^2^ = .04, indicating that the delaying effect of dual-tasking did not significantly affect the performance on the control blocks. Most importantly, the Triplet × Group interaction was not significant, *F*(1, 66) = 0.13, *p* = .72, η*_p_*^2^ = .002, revealing a lack of a statistically significant difference between groups in terms of the degree of probabilistic sequence knowledge also in the inserted control blocks.

# ASRT performance in the learning phase (matching criteria)

## Was the sequence knowledge of the two groups equal on Day 1?

To ensure that the two groups performed equally before the dual-task phase, we compared the performance of the two groups in the learning phase, between the two sessions, and in the beginning of the second session. First, we checked whether the two groups’ performance differed in the learning phase (Day 1). Reaction times were analyzed with a mixed-design ANOVA with the within-subject factor of Block (blocks 1-5 vs. blocks 6-10 vs. blocks 11-15 vs. blocks 16-20 vs. blocks 21-25 vs. blocks 26-30 vs. blocks 31-35 vs. blocks 36-40 vs. blocks 41-45) and the between-subjects factor of Group (dual-task group vs. single-task group).

The ANOVA of the learning scores revealed a significant main effect of Block, *F*(8, 528) = 17.92, *p* < .001, η*_p_*^2^ = .21, indicating that the degree of probabilistic sequence learning increased throughout the blocks. No group differences were found in the change of probabilistic sequence learning, Group: *F*(1, 66) = 0.47, *p* = .49, η*_p_*^2^ = .01. The dynamics of the change in the degree of sequence-specific learning also did not differ significantly between groups, Block × Group: *F*(8, 528) = 0.93, *p* = .50, η*_p_*^2^ = .01.

## Was the sequence knowledge equally consolidated in the two groups?

Next, we checked whether there was a significant difference in the level of consolidation between groups after the 24-hour offline period. To this end, we compared the performances between the last five blocks of the learning phase (learning phase blocks 41-45) and the first five blocks of the retrieval phase (retrieval phase blocks 1-5) with a mixed-design ANOVA with the within-subject factor of Block (learning phase blocks 41-45 vs. retrieval phase blocks 1-5) and the between-subjects factor of Group (dual-task group vs. single-task group). The performance at the beginning of the retrieval phase was also compared between groups with an independent samples t-test.

The ANOVA of the learning scores revealed a significant main effect of Block, *F*(1,66) = 8.58, *p* = .005, η*_p_*^2^ = 0.12, indicating that the degree of probabilistic sequence learning became smaller for the second session. No overall group differences emerged in the degree of probabilistic sequence learning, Group: *F*(1, 66) = 1.04, *p* = .31, η*_p_*^2^ = .02. The dynamics of the change in the degree of probabilistic sequence learning within blocks did not differ significantly between groups, Block × Group: *F*(1, 66) = 0.19, *p* = .66, η*_p_*^2^ = .003.

## Was the sequence knowledge equal before the dual-task phase on Day 2?

Finally, we compared the performance in the five blocks of the retrieval phase (retrieval phase blocks 1-5), to check whether there was a difference between groups before the dual-task phase. Independent samples t-test did not reveal significant difference between groups, *t*(66) = 1.16, *p* = .25.

# Analysis of the full sample

To ensure that our results were not due to the matching of groups, we repeated the analyses of the test and control blocks without excluding participants (*n* = 81).

## Did the retrieval of the sequence knowledge differ between groups in the test blocks?

First, we performed a mixed-design ANOVA on the learning scores of the test blocks with the within-subject factor of Block (retrieval phase blocks 6-10 vs. retrieval phase blocks 12-16 vs. retrieval phase blocks 18-22) and the between-subjects factor of Group (dual-task group vs. single-task group).

The main effect of Block was significant, *F*(2, 158) = 3.74, *p* = .03, η*_p_*^2^ = .05, indicating that the degree of probabilistic sequence learning became higher from the first to the second units of five blocks (*p* = .02), but remained stable afterwards (*p* = .88). Importantly, the main effect of Group did not reach significance, *F*(1, 79) = 0.009, *p* = .94, η*_p_*^2^ < .001, revealing a lack of statistically significant difference between groups in the degree of probabilistic sequence knowledge. This lack of difference did not change throughout the blocks, as revealed by a non-significant interaction between the Block and Group factors, *F*(2, 158) = 0.82, *p* = .44, η*_p_*^2^ = .01.

## Did the sequence knowledge of the two groups differ in the test blocks measured by the standardized scores?

We repeated the above analysis with the standardized learning scores (see Materials and methods section for description of the standardization process). The main effect of Block was significant, *F*(2, 158) = 4.81, *p* = .01, η*_p_*^2^ = .06, indicating that the degree of probabilistic sequence learning became higher from the first to the second units of five blocks (*p* = .01), but remained stable afterwards (*p* = .80). Importantly, the main effect of Group did not reach significance, *F*(1, 79) = 0.48, *p* = .49, η*_p_*^2^ = .006, indicating a lack of statistically significant difference between groups in the degree of probabilistic sequence knowledge. This similarity did not change throughout the blocks, as indicated by a lack of interaction between the Block and Group factors, *F*(2, 158) = 1.13, *p* = .33, η*_p_*^2^ = .01.

## Did the sequence knowledge of the two groups differ in the control blocks?

We also compared the sequence knowledge of the two groups in the control blocks. The independent samples t-test revealed no difference between groups, *t*(79) = 0.27, *p* = .79.

## How did the learning scores of the test blocks and the control blocks compare?

Next, we investigated if the learning scores of the test and control blocks differed and whether it was similar between the two groups. The Block Type × Group ANOVA on the learning scores did not reveal a significant main effect of Block Type, *F*(1,79) = 2.89, *p* = .09, η*_p_*^2^ = .04, suggesting a lack of significant difference in the measured sequence knowledge between the test and control blocks (i.e., between the periods where the stimulus stream contained colored stimuli). The main effect of Group did not reach significance, *F*(1, 79) = 0.38, *p* = .87, η*_p_*^2^ < .001, indicating that the two groups performed similarly in the retrieval phase. More importantly, there was no statistically significant difference between the two groups in how the learning scores developed between the two types of blocks, as suggested by the non-significant interaction of the Block Type and Group factors, *F*(1, 79) = 0.10, *p* = .75, η*_p_*^2^ < .001.

Taken together, similar results were obtained if we included all of the participants.

# Inclusion-exclusion task

We assessed the “*Process Dissociation Procedure (PDP)*” [1] by administering the Inclusion-Exclusion Task [2–5]. Employing this task, we revealed whether the participants gained explicit conscious knowledge about the probabilistic regularities during the ASRT task. Before administering the task (after completing the learning tasks), we informed the participants that the appearance of the stimuli followed a regularity. Then, in the first part of the task, we asked them to generate a sequence of button presses that follows the regularity of the ASRT task, using the same four response buttons they used during the ASRT task (*Inclusion condition*). They performed four runs of the Inclusion condition, and each run finished after 24 button presses, which is equal to three rounds of the eight-element alternating sequence [6–8]. After that, participants were asked to generate new sequences of responses that are *different* from the learned one (*Exclusion condition*). The Exclusion condition also contained four runs.

According to the PDP, participants can achieve successful performance in the Inclusion condition by solely implicit knowledge (explicit knowledge can also boost performance, but it is not necessary to the successful completion of the task). However, successful performance (i.e., intentionally generating *different* sequences) in the Exclusion condition can only occur if the participant has conscious knowledge about the learned probabilistic regularities (if the participant knows what to suppress). Generation of the learned probabilistic regularities above chance level even in the Exclusion task indicates that the participants rely on their implicit knowledge, as they cannot control the generation of the learned sequences consciously. To test whether the participants gained consciously accessible triplet knowledge, we calculated the percentage of producing high-probability triplets in the Inclusion and the Exclusion conditions separately. We tested whether it differs from the probability of generating them by chance. We also compared the percentages of generating high-probability triplets across conditions (Inclusion and Exclusion task) and groups (dual-task group and single-task group) (for more details about the Inclusion-Exclusion task, see: [6–8]).

## Analysis

To check whether the participants developed conscious knowledge about the learned probabilistic regularities, we compared the probability of high-probability triplets in the generated sequences in the Inclusion-Exclusion test to chance level (25%) with the help of one-samples t-tests, separately for the two conditions (Inclusion and Exclusion). We also compared the performance in the Inclusion and Exclusion conditions separately for the two groups with paired-sampled t-tests. Finally, we compared the percentages of generating high-probability triplets between the two groups with independent samples t-test for the Inclusion and Exclusion condition as well. The statistical analysis was also carried out by using IBM SPSS Statistics 25.

## Did the participants develop conscious knowledge about the probabilistic regularities and was it different between groups?

In the dual-task group, five participants were excluded from this analysis, as they were apparently not following the instruction properly in the Exclusion condition (they did not generate diverse sequences). The participants of the dual-task group generated 6.03% more high-probability triplets than chance level (25%) in the Inclusion condition, *t*(28) = 4.07, *p* < .001, and 4.45% more high-probability triplets in the Exclusion condition, *t*(28) = 2.31, *p* = .03. Comparing the two conditions, no significant difference was found, *t*(28) = 0.81, *p* = .43. In the single-task group, seven participants were excluded in total, as six participants did not follow the instructions in the Exclusion condition and one participant in the Inclusion condition. Overall, participants in this group generated 5.86% more high-probability triplets in the Inclusion condition than chance level, *t*(26) = 5.53, *p* < .001, and 4.67% more in the Exclusion condition, *t*(26) = 2.41, *p* = .02. Comparing the two conditions, no significant difference was found, *t*(26) = 0.60, *p* = .56. Comparing the performances of the two groups, no differences were found neither in the Inclusion, *t*(54) = 0.09, *p* = .93, nor in the Exclusion condition, *t*(54) = -0.08, *p* = .94. Taken together, the results suggest that both groups acquired the knowledge of the probabilistic regularities but could not consciously access and control it.

# References

1. Jacoby LL. A process dissociation framework: Separating automatic from intentional uses of memory. J Mem Lang. 1991;30: 513–541. doi:10.1016/0749-596X(91)90025-F

2. Destrebecqz A, Peigneux P, Laureys S, Degueldre C, Fiore G Del, Aerts J, et al. The neural correlates of implicit and explicit sequence learning: Interacting networks revealed by the process dissociation procedure. Learn Mem. 2005;12: 480–490. doi:10.1101/lm.95605

3. Destrebecqz A, Cleeremans A. Can sequence learning be implicit? New evidence with the process dissociation procedure. Psychon Bull Rev. 2001;8: 343–350. doi:10.3758/BF03196171

4. Jiménez L, Vaquero JMM, Lupiáñez J. Qualitative differences between implicit and explicit sequence learning. J Exp Psychol Learn Mem Cogn. 2006;32: 475–490. doi:10.1037/0278-7393.32.3.475

5. Fu Q, Dienes Z, Fu X. Can unconscious knowledge allow control in sequence learning? Conscious Cogn. 2010;19: 462–474. doi:10.1016/j.concog.2009.10.001

6. Kiss M, Nemeth D, Janacsek K. Stimulus presentation rates affect performance but not the acquired knowledge – Evidence from procedural learning. bioRxiv. 2019;650598: 650598. doi:10.1101/650598

7. Horvath K, Torok C, Pesthy O, Nemeth D, Janacsek K. Intention to learn differentially affects subprocesses of procedural learning and consolidation: Evidence from a probabilistic sequence learning task. bioRxiv. 2019; 433243. doi:10.1101/433243v2

8. Kóbor A, Janacsek K, Takács A, Nemeth D, Kobor A, Janacsek K, et al. Statistical learning leads to persistent memory: Evidence for one-year consolidation. Sci Rep. 2017;7: 1–10. doi:10.1038/s41598-017-00807-3
