# Supplementary figures and images for: Retrieval of a well-established skill is resistant to distraction: Evidence from an implicit probabilistic sequence learning task
Source: PLoS One. 2020 Dec 10;15(12):e0243541. doi: 10.1371/journal.pone.0243541 (PMC7728172; doi:10.1371/journal.pone.0243541)

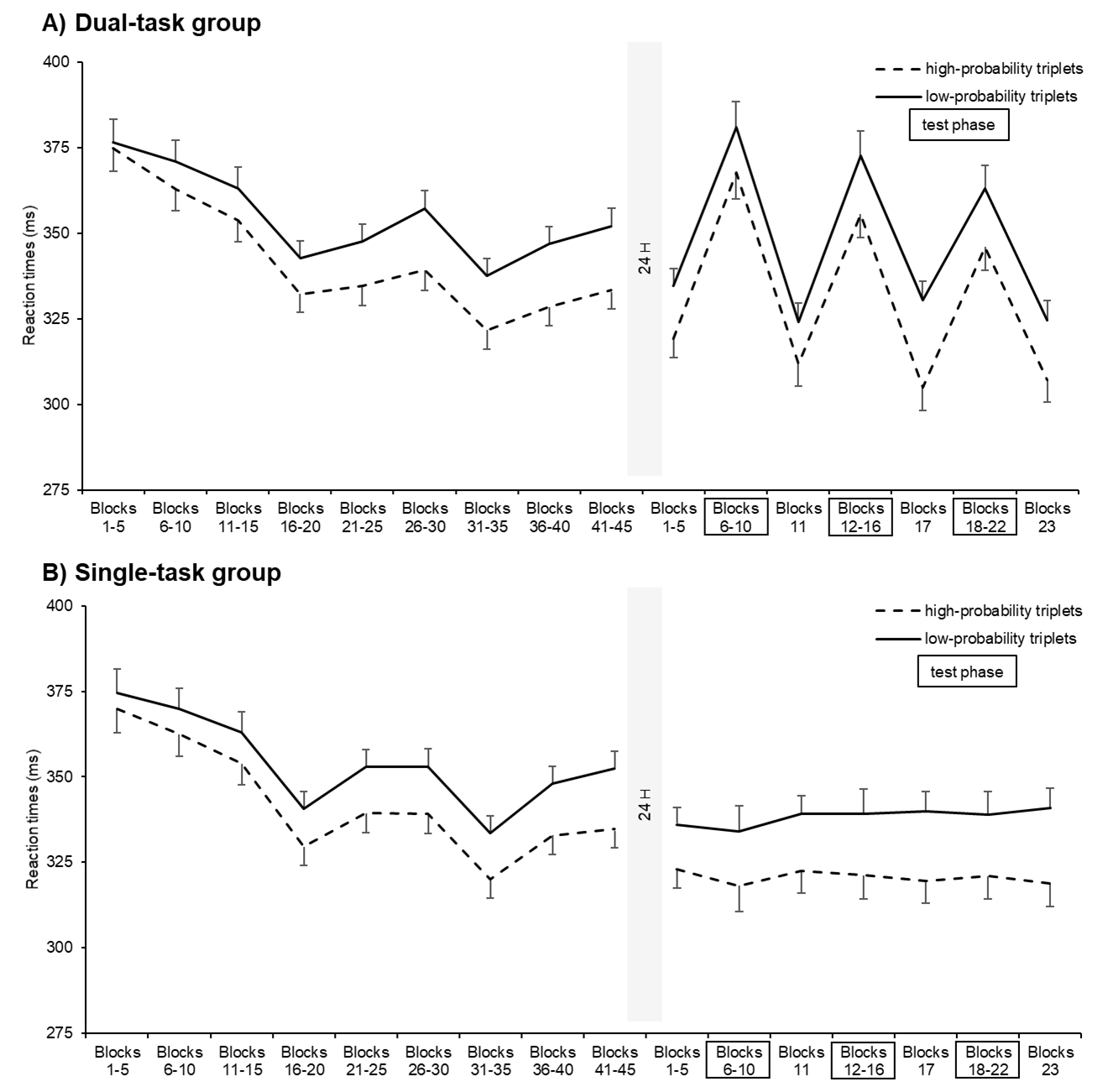

Supplement: S1 Fig — (TIF) [file pone.0243541.s002.tif]
