# Supplementary material for: Retrieval of a well-established skill is resistant to distraction: Evidence from an implicit probabilistic sequence learning task
Source: PLoS One. 2020 Dec 10;15(12):e0243541. doi: 10.1371/journal.pone.0243541 (PMC7728172; doi:10.1371/journal.pone.0243541)
Supplement: S1 Table — (DOCX) [file pone.0243541.s003.docx]

**Demographic characteristics of the full sample**

**S1 Table 1. Comparison of the two groups on age, years of education, handedness, working memory and short-term memory performance**

|  | *Dual-task group*  *M(SD)* | *Single-task group*  *M(SD)* | *Group comparison*  *(t-test results)* |
| --- | --- | --- | --- |
| Age (years) | 23.53 (4.40) | 23.07 (4.10) | *t*(79) = -0.48, *p* = .63 |
| Education (years) | 14.53 (2.23) | 14.88 (2.45) | *t*(79) = 0.66, *p* = .51 |
| Handedness (LQ) | 49.10 (37.10) | 38.00 (37.02) | *t*(79) = 1.35, *p* = .18 |
| Counting Span Score | 3.85 (.93) | 3.67 (.84) | *t*(79) = -0.89*, p* = .37 |
| Digit Span Score | 6.31 (1.12) | 6.07 (1.12) | *t*(79) = -0.99, *p* = .33 |
